# Wideband radiofrequency pulse sequence for evaluation of myocardial scar in patients with cardiac implantable devices

**DOI:** 10.3389/fradi.2024.1327406

**Published:** 2024-08-07

**Authors:** Neil D. Shah, Mayil Krishnam, Bharat Ambale Venkatesh, Fouzia Khan, Michele Smith, Darwin R. Jones, Patrick Koon, Xianglun Mao, Martin A. Janich, Anja C. S. Brau, Michael Salerno, Rajesh Dash, Frandics Chan, Phillip C. Yang

**Affiliations:** ^1^ Department of Medicine, Medical College of Wisconsin, Milwaukee, WI, United States; ^2^ Department of Radiology, Stanford University, Stanford, CA, United States; ^3^ Department of Medicine, Division of Cardiovascular Medicine, Johns Hopkins University, Baltimore, MD, United States; ^4^ Department of Medicine, Division of Cardiovascular Medicine, Stanford University, Stanford, CA, United States; ^5^ GE Healthcare, Menlo Park, CA, United States; ^6^ GE Healthcare, Munich, Germany

**Keywords:** wide band pulse, late gadolinium enhancement (LGE), cardiac MRI (CMR), intracardiac device, image quality (IQ)

## Abstract

**Background:**

Cardiac magnetic resonance is a useful clinical tool to identify late gadolinium enhancement in heart failure patients with implantable electronic devices. Identification of LGE in patients with CIED is limited by artifact, which can be improved with a wide band radiofrequency pulse sequence.

**Objective:**

The authors hypothesize that image quality of LGE images produced using wide-band pulse sequence in patients with devices is comparable to image quality produced using standard LGE sequences in patients without devices.

**Methods:**

Two independent readers reviewed LGE images of 16 patients with CIED and 7 patients without intracardiac devices to assess for image quality, device-related artifact, and presence of LGE using the American Society of Echocardiography/American Heart Association 17 segment model of the heart on a 4-point Likert scale. The mean and standard deviation for image quality and artifact rating were determined. Inter-observer reliability was determined by calculating Cohen's kappa coefficient. Statistical significance was determined by *T*-test as a *p* {less than or equal to} 0.05 with a 95% confidence interval.

**Results:**

All patients underwent CMR without any adverse events. Overall IQ of WB LGE images was significantly better in patients with devices compared to standard LGE in patients without devices (*p* = 0.001) with reduction in overall artifact rating (*p* = 0.05).

**Conclusion:**

Our study suggests wide-band pulse sequence for LGE can be applied safely to heart failure patients with devices in detection of LV myocardial scar while maintaining image quality, reducing artifact, and following routine imaging protocol after intravenous gadolinium contrast administration.

## Introduction

Cardiac magnetic resonance imaging (CMR) has been identified as a clinically valuable tool to identify late gadolinium enhancement (LGE) patterns, aiding in the diagnosis and treatment of myocardial diseases such as ischemic and non-ischemic cardiomyopathies, among others. Studies with LGE to detect myocardial scar and assess cardiac viability have demonstrated significant impact in patient care (1–6).

Patients with cardiomyopathies often have cardiac implantable electronic devices (CIED), which pose a technical challenge to standard LGE imaging sequences (7). Currently, more than 3 million Americans have CIEDs, including pacemakers, intracardiac defibrillators (ICDs), and cardiac resynchronization therapy (CRT) (8). As a result of the growing indications for pacemakers, ICDs and CRTs, the number of patients with such devices is anticipated to increase with a higher need for CMR within this population for accurate assessment of left ventricular ejection fraction (LVEF), LV volume, and myocardial scar. These assessments remain critical in clinical management to evaluate, manage, treat, intervene and/or prognosticate on patients with ischemic as well as non-ischemic cardiomyopathies.

Technical challenges in CMR of patients with CIEDs stems from local resonance frequency shifts as large as 2–6 kHz rendering standard imaging ineffective due to inadequate spectral bandwidth to overcome generator induced off-resonance artifacts (hyperintense bands, signal voids in myocardium). This difficulty results in non-diagnostic images especially for myocardial scar evaluation (9). Prior studies have demonstrated feasibility in wide band RF (WB) LGE pulse sequences in reducing imaging artifact related to ICDs both on 1.5 T and 3.0 T scanners (7, 10). Additionally, WB pulse LGE sequences were found to be safe for non-conditional MRI devices (11). Therefore, further clinical studies are warranted to validate the reliability of the wideband technique in routine clinical practice and to determine whether patients with ICDs can be safely and successfully imaged with current MRI techniques.

The purpose of our study was to evaluate the safety and image quality of the wide-band RF inversion-recovery pulse LGE sequence for detection of myocardial scar in heart failure patients with CIEDs and to compare the results with standard LGE sequence in heart failure patients without intracardiac devices.

## Methods

### Patient enrollment

Consecutive 23 heart failure (HF, LVEF <40%) patients who were enrolled in the Stem Cell Injection in Cancer Survivors (Seneca, NCT02509156) and Combination of mesenchymal and c-kit + Cardiac Stem cells as Regenerative Therapy for Heart Failure (Concert HF, NCT02501811) clinical trials underwent cardiac MRI for detection of myocardial scar over a two-year period.

### Image acquisition

Cardiac MRI was obtained on a 1.5 T Signa HDx TwinSpeed Scanner (GE HealthCare, Waukesha, WI) with retrospectively gated cine balanced Steady State Free Precession (bSSFP) of short axis and long axis planes, Look-Locker inversion recovery scout (Cine IR of GE) to select appropriate nulling time at 17 min post gadolinium contrast administration, and conventional post-contrast breath held T1-weighted inversion recovery gradient echo for LGE sequences of short and long axis planes for patients without CIEDs. Fast spoiled gradient echo sequence (fSPGR) for cine and wide-band radiofrequency inversion recovery pulse sequence for LGE sequences were utilized in heart failure patients with CIEDs.

CMR on patients with CIEDs was completed at total lung capacity at end-inspiration, as opposed to resting lung volume at end-expiration, to increase the distance between the device and the heart. Patients with CIEDs were also instructed to extend their arms above their head during the CMR exams, and the device was gently moved in the supero-lateral direction to further increase the device to heart distance.

Cine images in patients with devices were acquired using traditional fSPGR images. The frequency bandwidth was increased manually up to 500–1,000 Hz/pixel to reduce the metallic image artifacts. Cine images in patients without CIEDs were acquired using bSSFP sequence. CMR parameters for fSPGR cine images on patients with devices were the following: repetition time (TR) = 8–10 ms, echo time (TE) = 3–5 ms, flip angle (FA) = 20°, 8 mm slice thickness, 2 mm gap, flow compensation and image matrix = 256 × 128. CMR parameters for bSSFP cine images in patients without devices are the following: TR/TE = 3.8/1.6 ms, FA = 45°, 8 mm slice thickness, 2 mm gap, flow compensation and image matrix = 256 × 128.

**Figure 1:**
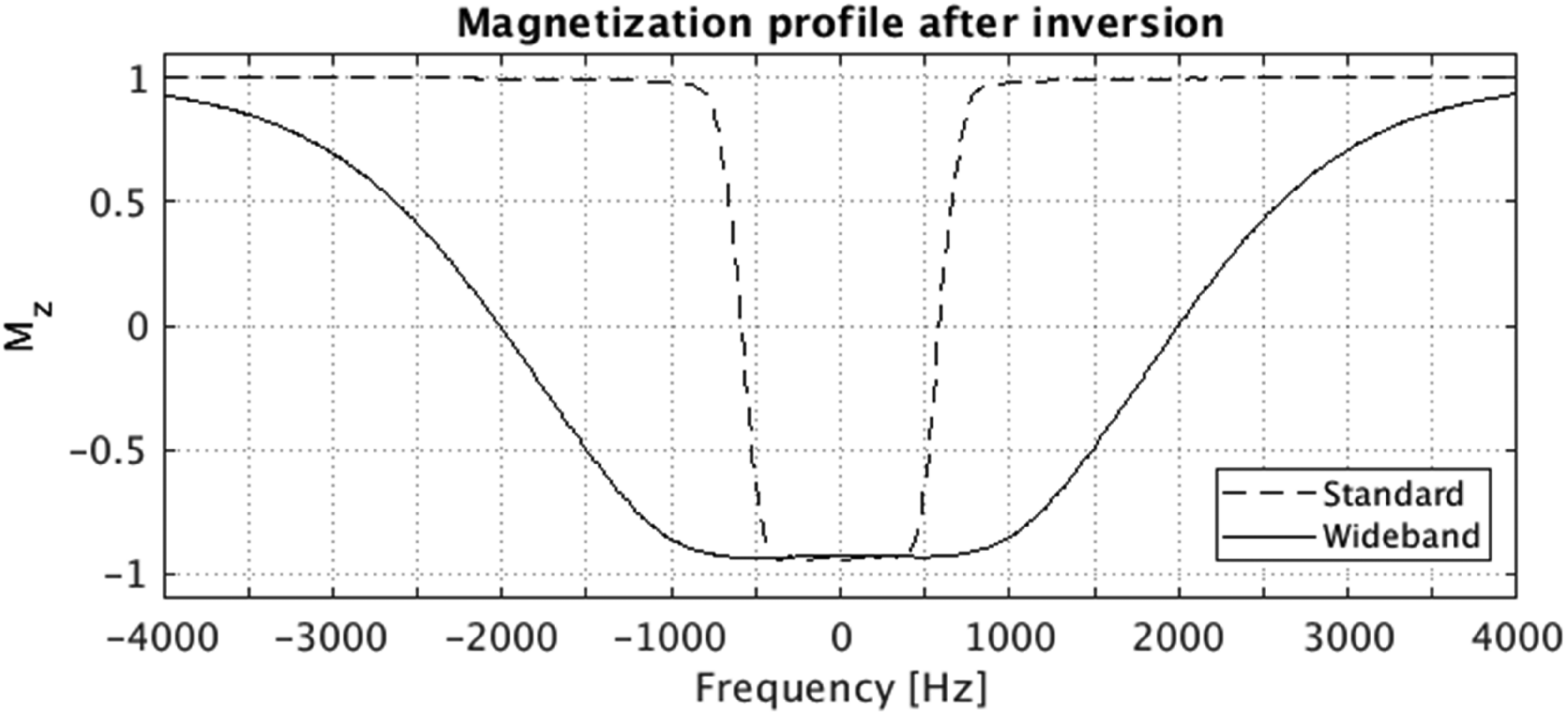
Bloch simulated magnetization profile after the standard (vendor product) and the wideband (proposed) adiabatic inversion RF pulse. The Bloch simulation was performed considering both T1 and T2 relaxation parameters (T1 = 1,015 ms, T2 = 52.2 ms), which are within the typical range for myocardial T1/T2 values. The FWHM bandwidth for standard and wideband pulse are 1,160 Hz and 4,020 Hz, respectively.

LGE images in patients with CIEDs were acquired 20 min after gadolinium contrast injection (Dotarem, 0.2 mmol/kg) using a modified WB inversion prepared segmented gradient-echo sequence consisting of the following: adiabatic full-passage inversion RF pulse design using tanh/tan modulation functions: pulse duration = 8 ms, B1 max amplitude = 12 microTesla, xi = 10, kappa = arctan(10), bandwidth FWHM = 4,020 Hz (7, 12) (Figure 1). In patients without CIEDs, LGE images were acquired using conventional inversion recovery segmented gradient echo sequence with CMR parameters that are similar to the WB-LGE sequence, except that pixel bandwidth was set to 100–150 Hz/pixel (13). CMR parameters for WB-LGE images were the following: TR = 4.1 ms, TE = 1.5 ms, field of view (FOV) = 360 mm, FA = 25°, readout bandwidth = 500 Hz/pixel, 8 mm slice thickness, 2 mm slice gap, spatial resolution = 1.4 × 1.9 mm, 1kHz adiabatic hyperbolic secant inversion RF pulse bandwidth (Figure 1), and inversion time (TI) = 250–400 msec. Both WB and standard sequences had 20 views per segment with 15 s of breath-holding.

The LGE images were acquired with the properly chosen TI (250–350 ms) to null the signal in myocardium and were reconstructed in both PSIR and magnitude. With the properly chosen TI time, the reviewers preferred doing the diagnosis using the magnitude images of LGE. The surface coil intensity correction was not used during the time that patients were scanned.

Patients with CIEDs were scanned under safe environment, following established protocol, including supervision of the procedures by cardiologist with CMR expertise and electrophysiology nursing support for device interrogation and monitoring pre and post cardiac MRI.

### Image interpretation

Two level-three CMR readers (one radiologist and one cardiologist with expert experience of over 15 years each) independently reviewed late gadolinium images of all heart failure patients. The reviewers were blinded to clinical data as well as to each other's interpretation.

A series of LGE sequences of twenty-three patients obtained over two-year period was analyzed for image quality and artifact on a 4-point Likert scale. Each segment of the AHA/ASE 17-segment model was analyzed for image quality and artifact, including device related artifact and nulling artifact and myocardial scar (14, 15).

Image quality was rated on a 4-point Likert scale as follows based upon the percentage of myocardium that was well evaluated: 1 = poor image quality (0%–25%), 2 = fair definition of myocardium, LGE reliably assessed (26%–50%), 3 = good definition of myocardium (51%–75%), and 4 = excellent definition of myocardium (76%–100%).

Artifact was assessed as overall and device related artifact. Common artifacts, which may be identified on LGE sequences, include respiratory artifact, cardiac motion from arrhythmia, nulling artifact related to inappropriate TI selection, and device related. Device related artifact was identified as signal loss or hyperintense band in an area where an intra-cardiac device was identified. Image artifacts from devices were evaluated by readers on a similar 4-point scale: 1-No artifact, excellent definition of anatomy to assess scar, 2- Minimal artifact but good definition of anatomy to assess myocardial scar, 3- Mild artifact but adequate definition of anatomy to assess myocardial scar, and 4- Moderate to severe artifact with poor definition of anatomy for confident assessment of LV myocardial scar. The readers also assessed for the presence or absence of myocardial scar.

### Statistics

The mean and standard deviation for image quality and artifact ratings were determined. Inter-observer reliability was determined by calculating Cohen's kappa coefficient (16). Statistical significance was defined as a *p* ≤ 0.05 with a 95% confidence interval.

## Results

LGE images on a total of 57 cardiac MRIs from 23 patients were reviewed for image quality, artifact, and myocardial scar. 16 of the 23 patients had intracardiac devices while 7 did not have a device. Clinical demographic data and types of devices are included in Tables 1, 2.

**Table 1:** Clinical demographics.

	Device	No device
Age	46–71	56–74
Gender	Male14, Female 2	Male 5 Female 2
Race/ethnicity	Hispanic/Latino 2, White 14	Asian 1, Hispanic 2, White
Comorbidities		
CAD	7	5
Hypertension	7	3
Hyperlipidemia	3	3
Congestive heart failure	7	0
Diabetes MELLITUS	5	2
COPD	0	1
Cerebrovascular disease	0	1
Cancer	5	3
Arrythmias		
Atrial Fibrillation	2	1
PVCs	1	2
Device Type		
Pacemaker	7 (with ICD)	
Transvenous ICD	0	
Subcutaneous ICD	2	
CRT	7	
Manufacturer		
Medtronic	Medtronic 12	
Boston scientific	Boston Sci 1	
St. Jude	St Jude 1	
Biotronik	Biotronik 2	

**Table 2:** Types of devices.

Medtronic DTBA 1D4 Viva XTCRT-D
St. Jude Medical/CD3369-40Q (Pacemaker + ICD)
Medtronic/viva XT CRT-D
Biotronik, 404633 Intica 7 VR-T DX ProMRI
Boston Scientific Teligen 100/E110 (Pacemaker + ICD)
Medtronic/Protecta XT DR (Pacemaker + ICD)
Medtronic, DDBB1D1 (Pacemaker + ICD)
Medtronic Evera MRI XT DR DDMB1D1
Medtronic/Model DVAB1D1 (Pacemaker + ICD)
Medtronic/Evera MRI XT DR DDMB1D4
Medtronic CRT-D/DT BA1QQ
Inventra 7 VR-T DX CRT-D
DVFBID4
Medtronic/DTMB1QQ Amplia MRI (Pacemaker + ICD)
Evera MRI XT DR DDMBID4
Evera MRI XT DR DDMBID4 (Pacemaker + ICD)

**Figure 2:**
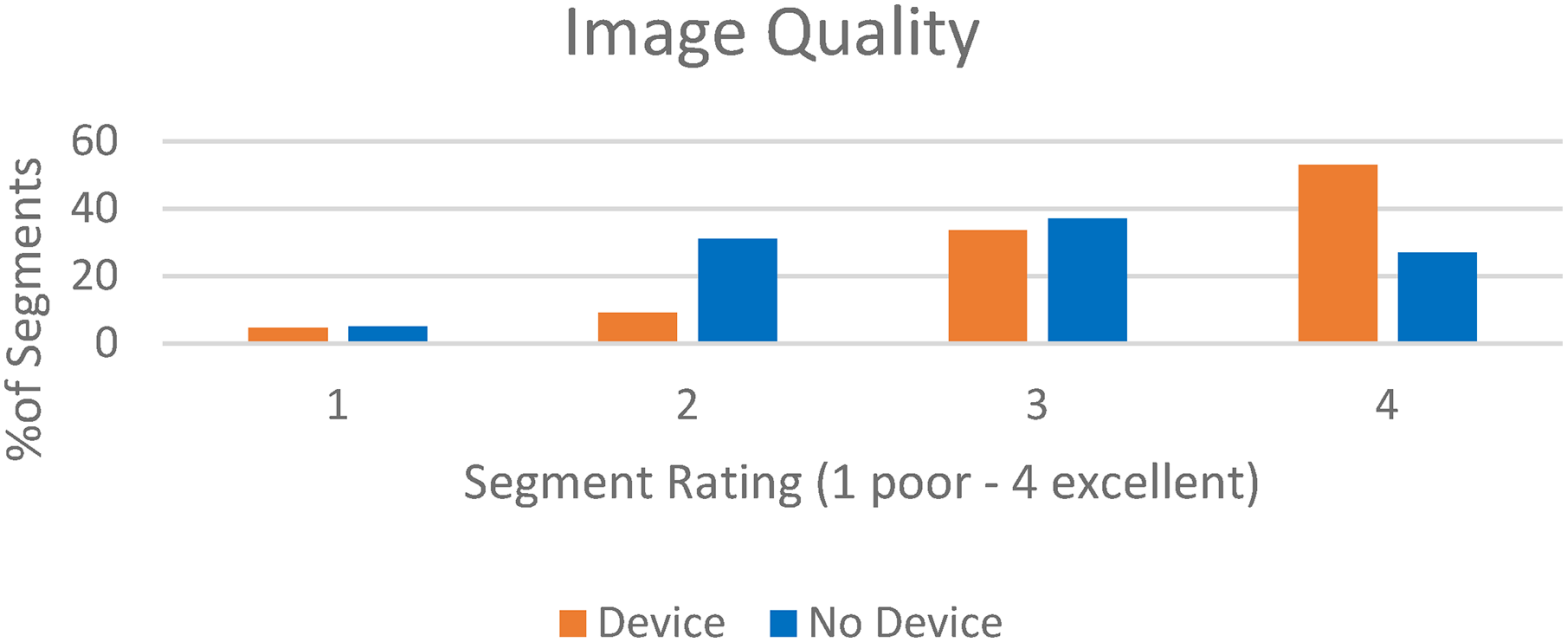
In central illustration—image quality rating in patients with intracardiac devices scanned with wideband LGE vs. patients without devices scanned with standard LGE.

**Figure 3:**
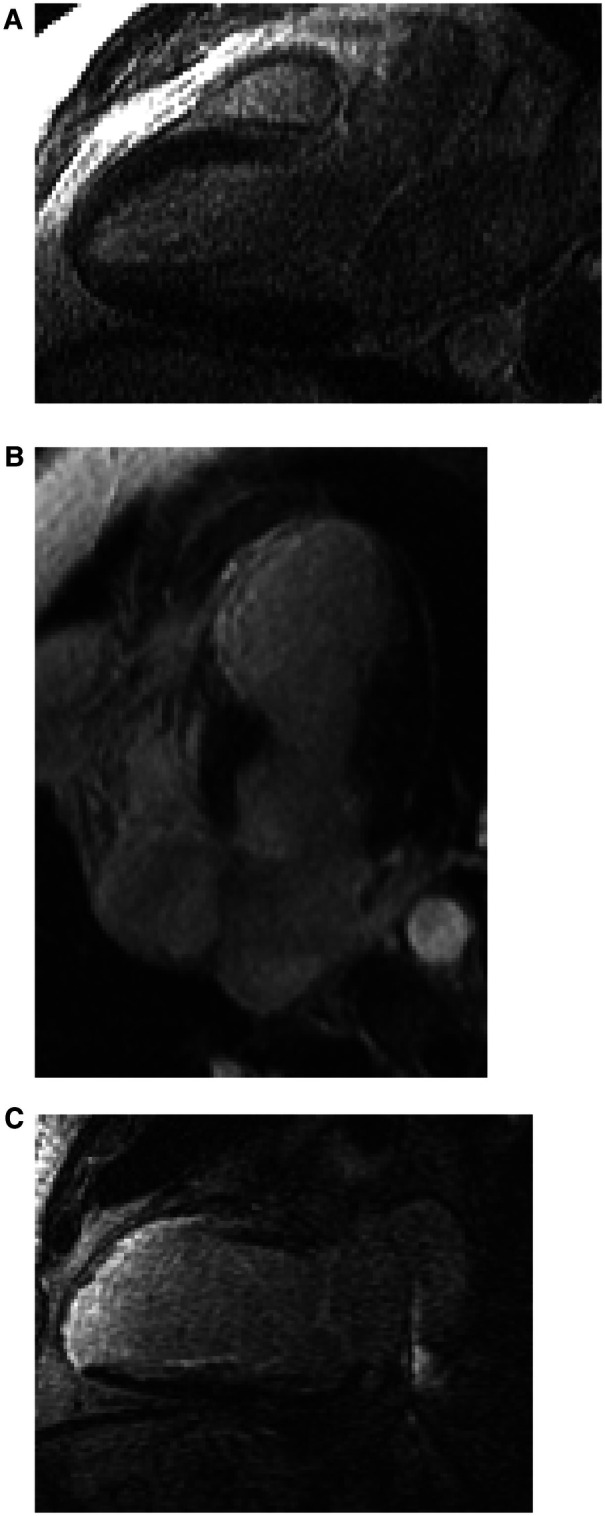
Representative images of LGE images of evaluated patients. (**A**) 3 chamber view in a patient without LGE or intracardiac device (**B**) 4 chamber view in a patient without intracardiac device but with LGE (**C**) 2 chamber view in a patient with scar and intracardiac device using wide-band sequence.

There were 40 MRI (680 segments) analyzed in patients with CIEDs with 17 scans (289 segments) analyzed in patients without CIEDs. 86% of the segments in patients with CIEDs received a rating of at least 3 (good image quality) compared to 64% of patients without devices (Figure 2). Approximately 5% of the segments in each subset of patients had an image quality rating of 1 (poor). Sample images are demonstrated in Figure 3.

The mean image quality rating in patients with CIEDs receiving the WB pulse sequence was 3.5 ± 0.8 and 2.9 ± 0.9 in patients without CIEDs (*p* = 0.001), suggesting IQ of WB LGE was good to excellent and statistically better than standard LGE. Overall artifact rating in patients who had CIEDs was 1.6 ± 0.9 while it was 1.8 ± 0.7 in patients without devices (*p* = 0.049), indicating that the WB LGE images were statistically better in reducing the artifact. Late gadolinium enhancement was identified in 45% of the patients with CIEDs in comparison to 29% of patients without CIEDs (Table 3).

**Table 3:** Central illustration (Figure 2 & Table 3, below).

Category	Device/WB LGE	No Device/LGE	*P*-value
Patients	16	7	
Number of segments (scans)	680 (40)	289 (17)	
Image Quality mean rating	3.5 ± 0.8	2.9 ± 0.9	0.001
Artifact	1.6 ± 0.9	1.8 ± 0.7	0.049
Device Artifact	1.1 ± 0.4		
LGE (%)	45%	29%	

Evaluating image quality and artifact in implantable electronic devices with wide band pulse sequence.

There were no adverse events such as inappropriate ICD shocks, pacemaker over-sensing, brady or tachyarrhythmia, or increased incidence of ventricular/atrial arrhythmia with the WB pulse or contrast reaction in either group. Additionally, the length of time for image acquisition was similar in both groups (Table 4).

**Table 4:** Time of image acquisition.

	Wide-band LGE	Standard LGE
Single-slice Acquisition	Single breath-hold, ∼15 s	Single breath-hold, ∼15 s
Full LGE (SAX stack, 4/3/2 chamber)	21 ± 5 min	20 ± 2 min
Whole CMR Exam	51 ± 5 min	49 ± 2 min

There was fair agreement between readers on image quality rating (56% agreement, Cohen’s kappa = 0.3) There was almost perfect agreement when segments were analyzed for the presence of scar (93% agreement, Cohen’s kappa = 0.85).

## Discussion

This study compared image quality and artifact from a wide band pulse sequence in heart failure patients with CIED to standard late gadolinium enhancement sequences in patients without intracardiac device.

Our study demonstrates superior image quality in patients undergoing wide band pulse sequence with a statistically significant reduction in overall artifact rating when compared to patients without CIED. Our results are reflected by reduction in noise with the wide-band pulse sequence, most likely due to an increased slice thickness and sub-optimal nulling of normal myocardium with the standard LGE images.

The feasibility of using the WB pulse sequence to identify LGE by comparing a modified WB pulse sequence in patients with intracardiac device to the standard LGE images on same patients using 1.5 T Siemens scanner has been demonstrated (7). Similar studies demonstrated feasibility of WB-LGE imaging on device patients using 3 T (10). These prior studies suggested a need for clinical studies to assess if WB LGE can be safely applied to all patients with cardiac devices. Image quality and artifact in patients with CIED have been studied previously as well (17). However, no prior studies have compared WB pulse sequence in patients with CIED to standard LGE sequences in patients without CIED in the HF patient population.

Our study is unique in that the WB pulse sequence group was compared to conventional LGE image acquisition while maintaining a uniform standard delay time following gadolinium contrast injection. In addition, we used a 1.5 T GE TwinSpeed scanner to demonstrate clinical utility of WB-LGE across multiple platforms. Additional strengths of our study include the number of myocardial segments analyzed over multiple years and from two different clinical trials with fair agreement between readers on image quality. Lastly, the number of segments analyzed in patients with devices were nearly triple those in patients without devices.

One disadvantage of the proposed sequence is that a wider bandwidth adiabatic radiofrequency pulse introduces a higher B1 amplitude, which will resulted in increased specific absorption rate (SAR). However, safe SAR levels are managed redundantly through both estimation during prescription and actual measurement during scanning. Additionally, SAR is typically low for LGE sequences. In this study, all LGE scans were performed within SAR limitations.

The limitations of the study included that this is a retrospective study without the ability to control for confounding factors. The results of our study are not generalizable to patients with subcutaneous ICDs as only two patients with such devices were included in this study and are limited by the patient sample size. Additionally, our results are limited to the identification of myocardial scar as we did not evaluate for type of myocardial scar or extent of scar. We were unable to compare conventional LGE sequences to WB-LGE in patients with devices as conventional imaging was not obtained in the device patients. Lastly, there was sub-optimal nulling and increased noise with conventional imaging, which may be related to small sample size as well as patient body habitus.

## Conclusion

Our study indicates that the use of the WB RF pulse sequence for LGE in HF patients with CIEDs is a safe approach that may accurately identify LV myocardial scar while also preserving image quality, minimizing artifact, and allowing routine imaging protocol after intravenous gadolinium contrast administration. There remains a significant clinical need for identification of scar in patients with devices, including determining the etiology of cardiomyopathy as well as planning for percutaneous and surgical interventions. Further prospective studies with a larger patient population are warranted to confirm the results of this study as well as identify the type and extent of myocardial scar using WB-LGE in this complex heart failure patient population with significant mortality and morbidity.

## Clinical Perspective

- Wide-band RF inversion recovery LGE imaging can be safely performed in heart failure patients (high risk) with CIED for reliable detection of LV myocardial scar.

- Wide-band RF inversion recovery LGE imaging provides better image quality and less artifact than the standard LGE imaging (no wide-band).

- LV myocardial scar can be reliably depicted by wide-band RF IR pulse LGE sequence, which can be safely performed in high-risk heart failure patients with implantable cardiac pacemaker, cardiac resynchronization, and defibrillator devices.

- This capability enables clinical diagnosis and prognosis in heart failure patients with CIED.

## Data Availability

The raw data supporting the conclusions of this article will be made available by the authors, without undue reservation.
